# Validation of a 5‐Year Prognostic Model for Parkinson's Disease

**DOI:** 10.1002/mdc3.14215

**Published:** 2024-09-29

**Authors:** Joana Ribeiro, Marta Camacho, Kirsten M. Scott, Julia C. Greenland, Jonathan R. Evans, David P. Breen, Ruwani S. Wijeyekoon, Roger A. Barker, Caroline H. Williams‐Gray

**Affiliations:** ^1^ Department of Clinical Neurosciences University of Cambridge Cambridge UK; ^2^ Nottingham University Hospital NHS Trust Nottingham UK; ^3^ Centre for Clinical Brain Sciences University of Edinburgh Edinburgh UK; ^4^ Anne Rowling Regenerative Neurology Clinic University of Edinburgh Edinburgh UK; ^5^ Usher Institute of Population Health Sciences and Informatics University of Edinburgh Edinburgh UK; ^6^ Cambridge Stem Cell Institute University of Cambridge Cambridge UK

**Keywords:** neuroepidemiology, Parkinson's disease, prognostic model

## Abstract

**Background:**

A simple prognostic model was previously developed to predict the probability of recently‐diagnosed patients reaching negative outcomes (postural instability, dementia or death) in a 5‐year period.

**Objectives:**

To validate this model in an independent cohort and establish utility at later time points.

**Methods:**

Validation was performed using data collected in an incident cohort at baseline, 2 and 4 years. Predicted negative outcome probabilities were compared to actual 5‐year outcomes.

**Results:**

The model, based on age, MDS‐UPDRS axial score and 60‐second animal fluency, predicted poor 5‐year outcome when applied at baseline, (area under the curve (AUC) 0.80), 2 years (AUC 0.82) and 4 years (AUC 0.71). Power calculations showed that selecting a subgroup with prognostic score >0.5 reduced the sample size required for a disease‐modifying trial.

**Conclusions:**

This 5‐year prognostic model has good accuracy when employed up to 4 years from diagnosis and may help stratification for disease‐modifying trials.

Parkinson's disease (PD) is highly variable in terms of rate of progression to the major milestones of postural instability (Hoehn and Yahr 3 or above), dementia and death.^1^ A longitudinal population‐based cohort study showed that median time to any unfavorable outcome was 3.8 years.^1^ However, the timing of these milestones in individual patients is difficult to predict, which is problematic in terms of counseling patients and planning long‐term management. Furthermore, this heterogeneity presents a challenge for clinical trials of putative disease modifying therapies, in which a treatment effect can be difficult to demonstrate in patients with a slowly progressive phenotype.^2^

We have previously developed (with Velseboer and colleagues) a prognostic model to predict the probability of patients with newly‐diagnosed PD reaching an unfavorable clinical outcome at 5 years from baseline.^3^ The logistic regression model was based on three simple clinical measures: age, UPDRS axial score and animal fluency in 60 seconds. It was externally validated in an independent cohort, which confirmed good discriminative ability between favorable and unfavorable outcomes, with an area under the receiver operating characteristic (ROC) curve of 0.85.^3^ However, this model has not been validated for use at a later timepoint after diagnosis, which would have significant real‐world value. Establishing whether this easily applicable model has utility for predicting 5‐year favorable/unfavorable outcomes for assessments performed at later timepoints would be informative for clinicians and may help inform recruitment of participants with a more rapidly progressing phenotype for disease‐modification trials.

## Methods

This study was performed using data from Parkinsonism: Incidence and Cognitive Heterogeneity in Cambridgeshire (PICNICS), an incident cohort study following patients meeting UKPDS Brain Bank criteria for PD from baseline (mean 0.24 years from diagnosis) with repeated assessments every 18–24 months. The prognostic model was applied using data collected at three timepoints—baseline (visit 1), and approximately 2 and 4 years after diagnosis (visits 2 and 3, respectively). The variables age, MDS‐UPDRS axial score and 60‐second animal fluency score at each visit were used in the prognostic calculator^4^ to estimate probability of reaching an unfavorable outcome (postural instability, dementia, or death) at 5 years. MDS‐UPDRS axial score was calculated in the ON state as the sum of MDS‐UPDRS items 3.9, 3.10, 3.12 and 3.13^5^ (equivalent to the UPDRS items used in the original model^3^).

Actual outcomes were determined using prospective follow‐up. In accordance with the original study, patients who reached postural instability, developed dementia or died in the 5‐year follow‐up period following application of the model were considered to have met unfavorable outcomes. All other patients were included in the favorable outcome group. Dementia diagnosis was determined using the MDS dementia criteria^6^ and confirmed by a neurologist. Date of dementia diagnosis was calculated as the midpoint between the visit at which diagnostic criteria were met, and the preceding visit. Postural instability was determined as reaching Hoehn and Yahr Stage 3 or higher, with the date of postural instability calculated as the midpoint between the visit at which it was first recorded, and the preceding visit. Medical records were screened for information on disease stage, dementia status and mortality if a participant was lost to follow‐up.

Participants who had already developed an unfavorable outcome at the initial timepoint for the modeling were excluded. Those lost to follow‐up before the 5‐year endpoint were also excluded unless they had already reached unfavorable outcomes prior to drop‐out. ROC curves were produced to assess model performance at visits 1, 2 and 3.

To investigate the possibility of selective attrition, we compared mean age, sex, MDS‐UPDRS total score, Revised Addenbrooke's Cognitive Examination^7^ score and time from PD diagnosis with Mann–Whitney *U* tests in participants who remained active in the study at the 5‐year timepoints versus those who were lost to follow‐up. All statistical analyses were performed using SPSS version 29.0.1 (IBM Corp., Armonk, NY, USA) and a significance threshold of *P* < 0.05 was used.

Finally, we performed power calculations to estimate sample sizes for a clinical trial, comparing inclusion of the entire sample versus selecting for participants with a prognostic score over 0.5 (50% probability of a negative outcome at 5 years), assuming a significance level of 0.05 and power of 80%. We selected the sum of MDS‐UPDRS parts I + II score as the outcome measure (recently recommended based on consensus clinician and patient input for disease modification trials in PD)^8^ and calculated mean change per year between visits 2 and 3. Data from visit 2 to 3 was used to avoid the confounding effect of the introduction of PD medication on disease progression scores, since only 57% of patients were on PD medication at visit 1, compared to 91% at visit 2. We used a sample size calculator^9^ to predict the required sample size to detect an effect equivalent to the difference between the natural annualized increase in MDS‐UPDRS parts I + II score (untreated group) and stable scores (disease modifying therapy group).

## Results

Out of 280 participants in the PICNICS cohort, 198 were included in the analysis at visit 1 (mean 0.26 years from diagnosis), 133 at visit 2 (mean 2.05 years from diagnosis), and 78 at visit 3 (mean 3.73 years from diagnosis); Figure S1. There were no significant differences in demographic and clinical characteristics between included participants and those excluded because of loss to follow‐up without known outcomes, except for a higher mean MDS‐UPDRS total score in the excluded group at visit 1 (4.53 points difference, *P* = 0.04); Table S1.

**Figure 1:**
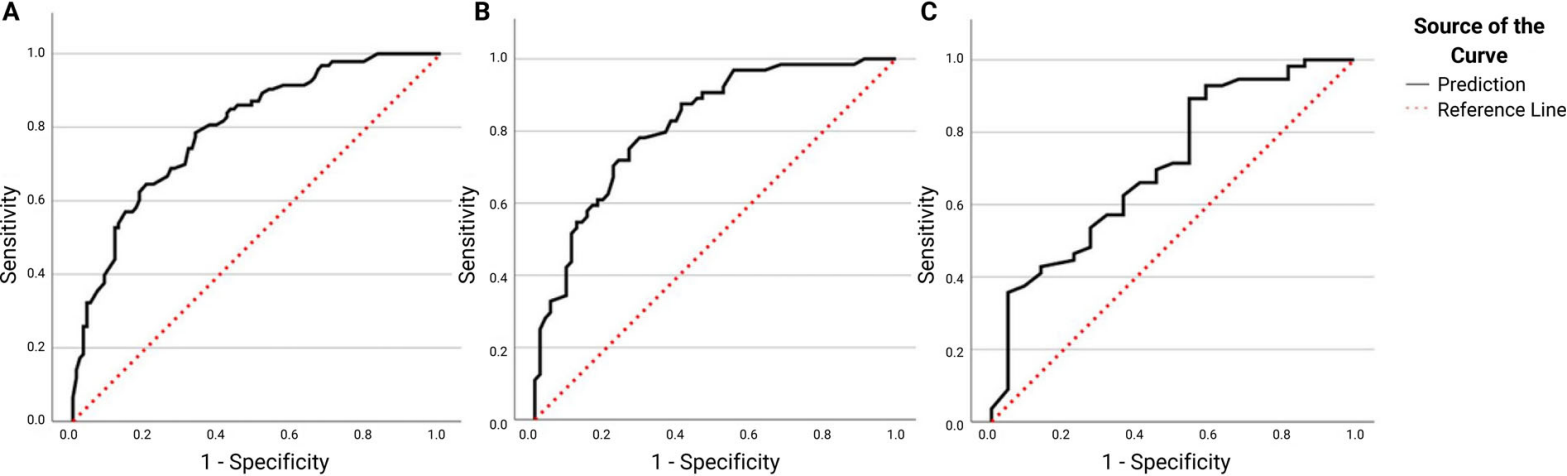
Receiving operator characteristic (ROC) curves for visit 1 at baseline (A), visit 2 at 2 years (B) and visit 3 at 4 years (C). The dotted red line indicates an area under the curve (AUC) of 0.5, representing a non‐informative model.

Unfavorable outcomes were reached in 93 (46.97%) participants at 5 years post visit 1, of whom 73 (36.87%) developed postural instability, 25 (12.63%) developed dementia, and 40 (20.20%) died (with some participants developing more than one negative outcome within 5 years). Following visit 2, 64 (48.12%) participants reached negative outcomes within 5 years, of whom 51 (38.35%) developed postural instability, 34 (25.56%) developed dementia and 48 (36.09%) died. Following visit 3, 56 (71.79%) participants reached negative outcomes within 5 years, of whom 51 (65.38%) developed postural instability, 23 (29.49%) developed dementia and 25 (32.05%) died. Area under the curve for visit 1, 2 and 3 models was 0.80 (0.74–0.86 95% CI, 0.03 SE), 0.82 (0.75–0.89 95% CI, 0.04 SE) and 0.71 (0.58–0.84 95% CI, 0.07 SE), respectively (Fig. 1).

Sample size calculations suggested that 294 participants would be required to detect a stabilization compared to the observed increase in MDS‐UPDRS I + II scores over 12 months, at power 80% and significance level 0.05 in a disease‐modifying clinical trial with 1:1 randomization to active drug or placebo, if selection was random. In contrast only 208 participants would be required if selected based on a prognostic score of over 0.5 (Table 1).

**Table 1:** Sample size calculations for a 12‐month randomized controlled trial of a disease modifying agent in an unstratified cohort versus a cohort with prognostic score >0.5

Prognostic score	Baseline MDS‐UPDRS I + II; mean (SD)	MDS‐UPDRS I + II change/year	Power (%)	Significance	Sample size
All	18.91 (9.36)	3.05	80	0.05	296
>0.5	21.62 (9.29)	3.59	80	0.05	208

Abbreviations: MDS‐UPDRS, Movement Disorders Society—Unified Parkinson's Disease Score; SD, standard deviation.

## Discussion

Our study has further validated the Velseboer et al^3^ PD prognostic model for use in newly‐diagnosed patients and demonstrates that it performs with good accuracy when applied several years after diagnosis. This freely‐available model, which is based on simple clinical parameters, can easily be applied in clinic and our findings further validate its real‐world utility.

This study used data from an incident cohort, which tracked PD progression over time using similar clinical variables and outcome measures as the cohorts employed in the previous study.^3^ It was therefore possible to directly apply the original model in this cohort at different time‐points. In addition to providing longer‐term validation, this study has provided additional external validation of this prognostic model in an independent cohort, with a larger sample size for both visit 1 and 2 assessments than the original study (n = 111 for model development, n = 108 for model validation). This confirms the utility of the model for prognostication and expands its application to data from follow‐up assessments. Furthermore, we have shown that application of this prognostic score allows for a reduction in the required sample size for a disease‐modifying clinical trial with the MDS‐UPDRS parts I + II as the outcome measure. While other predictive tools for PD progression have also been developed, they often require more complex clinical and genetic data to be collected, and/or predict specific motor or cognitive outcomes only, which limits their accessibility and clinical use.^10^, ^11^, ^12^, ^13^ As such, this type of stratification may prove invaluable in some of the newer multi‐arm trials that are being planned in PD^14^.

The decline in performance of the model at visit 3 likely reflects reduced sample size, as many patients had already achieved an unfavorable outcome by that assessment or were lost to follow‐up during the following 5‐year period. The reduced sample size also limits the robustness of our findings at this timepoint. Larger cohorts will be needed to allow testing of the model at later timepoints. However, a limitation of analysis in later stage patients is that aging would lead to a naturally increased effect of the variable “age” on the prognostic score, since mortality would be more prevalent, irrespective of PD progression. This concern did not apply to our study since mean age remained below the average life expectancy in the UK for all time‐points.^15^ Greater selective attrition of patients with more severe disease may also be an important limitation in a more advanced PD cohort. A further limitation of our analysis was the exclusion of participants who were lost to follow‐up with unknown outcomes prior to 5 years. Although this approach may have introduced bias if those lost to follow‐up were more likely to have a poor outcome, we considered this was preferable to including these participants with the assumption of a positive outcome. Furthermore, comparison of included participants with those lost to follow‐up showed that they were well‐matched in terms of baseline prognostic scores and most clinical variables (Table S1), suggesting selective attrition was unlikely to be a significant issue. Finally, whilst the simplicity of this composite model has great clinical value, it does not address the fact that dementia and postural instability might have different underlying pathologies. In the future, it would be interesting to develop models to predict these outcomes individually.

In conclusion, this study shows that a PD prognostic model previously developed for use at diagnosis^3^ also has utility in predicting 5‐year outcomes in people who have had PD for up to 4 years, although its performance is best at early stages. This remains an easily translatable tool for clinical use and may be helpful for management planning as well as for stratification to increase power and/or reduce sample sizes required for future clinical trials.

## Author Roles

(1) Research Project: A. Conception, B. Organization, C. Execution; (2) Statistical Analysis: A. Design, B. Execution, C. Review and Critique; (3) Manuscript Preparation: A. Writing of the first draft, B. Review and Critique.

J.R.: 1C, 2B, 3A.

M.C.: 1A, 1B, 1C, 2C, 3B.

K.M.S.: 1C, 2C, 3B.

J.C.G.: 1C, 3B.

J.R.E.: 1C, 3B.

D.P.B.: 1C, 3B.

R.S.W.: 1C, 3B.

R.A.B.: 1A, 3B.

C.H.W.‐G.: 1A, 1B, 1C, 2A, 2C, 3B.

## Disclosures

**Ethical Compliance Statement:** All patients provided written consent. The study was approved by Essex 2 Research Committee (REC reference number 07/M0302/138). We confirm that we have read the Journal's position on issues involved in ethical publication and affirm that this work is consistent with those guidelines. For the purpose of open access, the author has applied a Creative Commons Attribution (CC BY) license to any Author Accepted Manuscript version arising from this submission.

**Funding Sources and Conflicts of Interest:** The PICNICS study has received funding from the Cure Parkinson's Trust, the Van Geest Foundation, the Medical Research Council (MRC) and Parkinson's UK. This work was also supported by the National Institute for Health Research (NIHR) Cambridge Biomedical Research Centre (NIHR203312). The views expressed are those of the author(s) and not necessarily those of the NIHR or the Department of Health and Social Care. M.C. was supported by Centre for Parkinson‐Plus. R.A.B. was supported by the Wellcome‐MRC‐Stem Cell Institute (Cambridge 203151/Z/16/Z). C.H.W.G. is supported by the MRC (MR/W029235/1) and the (NIHR) Cambridge Biomedical Research Centre (NIHR203312). K.M. Scott is currently an employee of AstraZeneca but was involved in this work prior to that employment. The authors declare that there are no additional disclosures to report.

**Financial Disclosures for the Previous 12 Months:** The authors declare that there are no additional disclosures to report.

## Supporting information


**Figure S1.** Participant inclusion and exclusion. Participants were excluded if they had missing baseline data, already met outcomes at that visit or had been lost to follow‐up without known outcomes during the 5‐year period.

**Table S1.** Demographic and clinical characteristics of participants who were included versus those who were lost to follow‐up with unknown outcomes.

## Data Availability

The data that support the findings of this study are available on request from the corresponding author. The data are not publicly available due to privacy or ethical restrictions.
