# Supplementary figures and images for: Validation of a 5‐Year Prognostic Model for Parkinson's Disease
Source: Mov Disord Clin Pract. 2024 Sep 29;11(11):1441–4. doi: 10.1002/mdc3.14215 (PMC11542283; doi:10.1002/mdc3.14215)

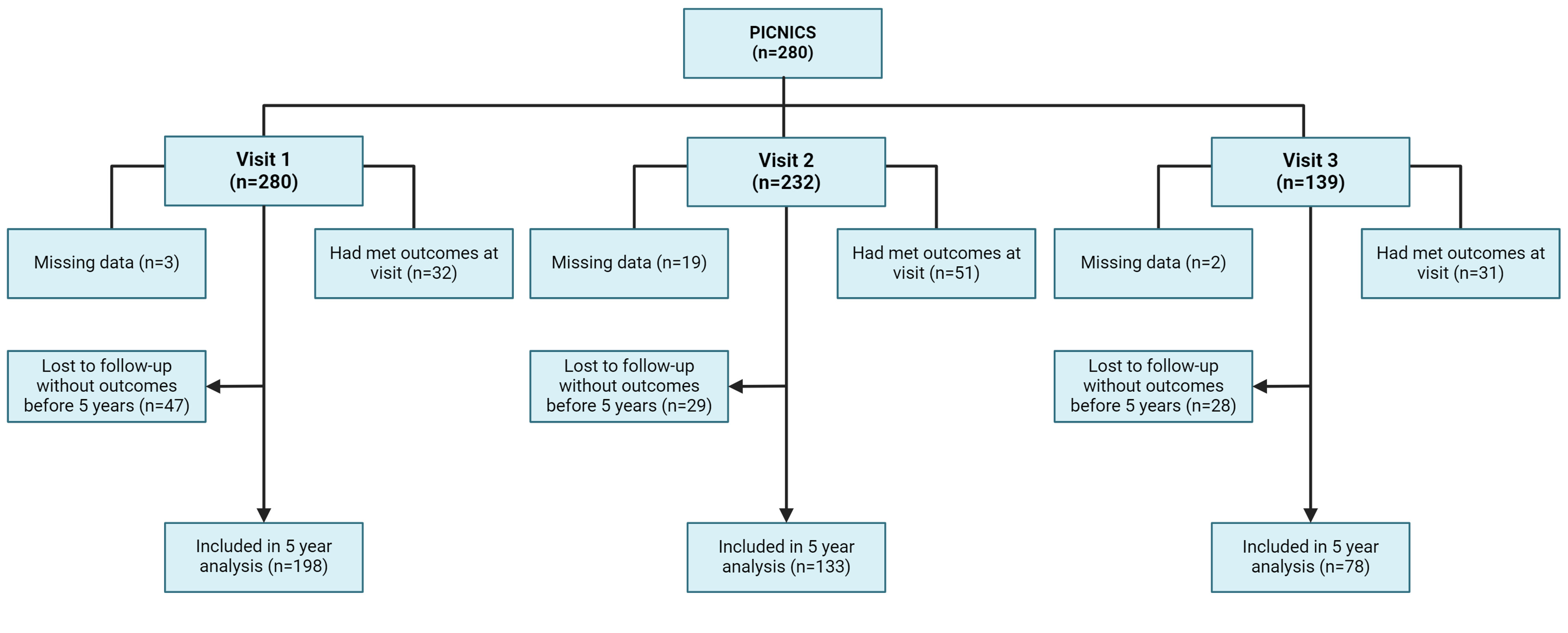

Supplement: Supplementary file 1 — Figure S1. Participant inclusion and exclusion. Participants were excluded if they had missing baseline data, already met outcomes at that visit or had been lost to follow‐up without known outcomes during the 5‐year period. [file MDC3-11-1441-s002.tif]
