# Supplementary material for: Validation of a 5‐Year Prognostic Model for Parkinson's Disease
Source: Mov Disord Clin Pract. 2024 Sep 29;11(11):1441–4. doi: 10.1002/mdc3.14215 (PMC11542283; doi:10.1002/mdc3.14215)
Supplement: Supplementary file 2 — Table S1. Demographic and clinical characteristics of participants who were included versus those who were lost to follow‐up with unknown outcomes. [file MDC3-11-1441-s001.pdf]

	Age	N (%) Male	MDS-UPDRS	ACE-R	Years since PD Diagnosis	Prognostic Scores
**Visit 1**						
Included (n=198)	67.88 (9.11)	131 (66.16)	43.34 (15.32)	90.72 (5.85)	0.26 (0.31)	0.59 (0.25)
LFU (n=47)	67.97 (10.48)	29 (61.7)	47.87 (16.53)	89.51 (7.11)	0.24 (0.37)	0.59 (0.27)
P-Value	0.87	0.56	0.04*	0.38	0.15	0.95
**Visit 2**						
Included (n=133)	68.35 (8.04)	89 (66.92)	48.85 (18.45)	91.25 (5.80)	2.05 (0.48)	0.60 (0.25)
LFU (n=29)	66.66 (9.81)	20 (68.97)	49.27 (4.91)	91.24 (4.91)	2.14 (0.57)	0.57 (0.24)
P-Value	0.33	0.83	0.71	0.81	0.62	0.50
**Visit 3**						
Included (n=78)	68.93 (8.66)	52 (66.67)	49.39 (20.93)	90.17 (7.21)	3.73 (0.59)	0.67 (0.24)
LFU (n=28)	71.39 (8.39)	20 (71.43)	53.78 (14.10)	91.61 (6.07)	3.7 (0.64)	0.63 (0.26)
P-Value	0.24	0.65	0.21	0.44	0.67	0.43
